# Opportunities for diversifying and enriching our article mix

**DOI:** 10.1186/s13584-020-00427-9

**Published:** 2020-12-03

**Authors:** Bruce Rosen, Stephen C. Schoenbaum, Avi Israeli

**Affiliations:** 1 Myers-JDC-Brookdale Institute, Jerusalem, Israel; 2 Josiah Macy Jr. Foundation, New York, NY USA; 3 Hebrew University Hadassah Medical School, Jerusalem, Israel; 4 Ministry of Health, Jerusalem, Israel

## Abstract

As 2020 comes to a close, the *Israel Journal of Health Policy Research* (IJHPR) will soon be starting its tenth year of publication. This editorial compares data from 2012 (the journal’s first year of publication) and 2019 (the journal’s most recent full year of publication), regarding the journal’s mix of article types, topics, data sources and methods, with further drill-downs regarding 2019.

The analysis revealed several encouraging findings, including a broad and changing mix of topics covered. However, the analysis also revealed several findings that are less encouraging, including the limited number of articles which assessed national policy changes, examined changes over time, and/or made secondary use of large-scale survey data. These findings apparently reflect, to some extent, the mix of studies being carried out by Israeli health services researchers.

As the senior editors of the IJHPR we are interested in working with funders, academic institutions, the owners and principal users of relevant administrative databases, and individual scholars to further understand the factors influencing the mix of research being carried out, and subsequently published, by Israel’s health services research community. This deeper understanding could then be used to develop a joint plan to diversify and enrich health services research and health policy analysis in Israel. The plan should include a policy of ensuring improved access to data, to properly support information-based research.

## Introduction

As 2020 comes to a close, the *Israel Journal of Health Policy Research* (IJHPR) will soon be starting its tenth year of publication. Its impact factor has increased from 1.25 in 2013 to 1.76 in 2020,^1^ and the number of submissions continues to climb. In 2019, the IJHPR accounted for 49% of the articles by Israeli authors in the Social Science Index’s Health Policy and Services category and 21% of such articles in the Public, Environmental, and Occupational Health category of that index.

As indicated in the journal’s mission statement, “(The) *Israel Journal of Health Policy Research* seeks to promote intensive intellectual interactions among scholars and practitioners from Israel and other countries regarding all aspects of health policy, health services research, (and) public health … in Israel. The aim of these intellectual interactions is to contribute to the development of health policy in Israel and around the world.” True to its mission, the journal is regularly drawing submissions from a wide variety of disciplines and a very broad range of Israeli universities, hospitals, health plans, and government agencies, as well as from leading institutions abroad^2^ [2].

As the journal’s senior editors, we sense that now is an appropriate time to analyze how the journal’s content has changed over time and to reflect on what changes we would like to see in the years ahead.

### Comparison of articles published in 2012 and 2019

Tables 1 and 2 present comparative data for 2012 (our first year of publication) and 2019 (the most recent full year of publication). Table 1 presents data on the overall^3^number of articles published and their mix in terms of topics and article types. Table 2, which focuses on original research articles, presents comparative data on data sources and key aspects of study methods.

**Table 1:** The IJHPR’s mix of articles types and topics, 2012 and 2019

	2012		2019	
	N	%	N	%
**Number of articles**	52	100%	85	100%
**Article types**				
Original research articles	17	33%	40	47%
Integrative articles	7	13%	11	13%
Commentaries	25	48%	28	33%
Other (editorials, meeting reports, etc.)	3	6%	6	7%
**Most common topics (topics with at least 5 articles in either year)**				
Quality of care	9	17%	5	6%
Health promotion / disease prevention	8	15%	15	18%
Health care workforce	8	15%	12	14%
Health disparities	0	0%	11	13%
Mental health (aside from dementia)	2	4%	9	11%
Dementia care (from a conference)	0	0%	7	8%
Staff-patient interactions	5	10%	2	2%
Digital health	2	4%	5	6%

**Table 2:** Data sources and methods for original research articles, 2012 and 2019

	2012		2019	
	N	%	N	%
**Number of original research articles**	**17**	**100%**	**40**	**100%**
**Data source**				
Database	5	29%	13	33%
Survey	8	47%	17	43%
Both database and survey	1	6%	4	10%
Other	3	18%	5	13%
**Secondary analysis of existing data**	6	35%	17	43%
**Examined changes over time**	4	24%	5	13%

**Fig. 1:**
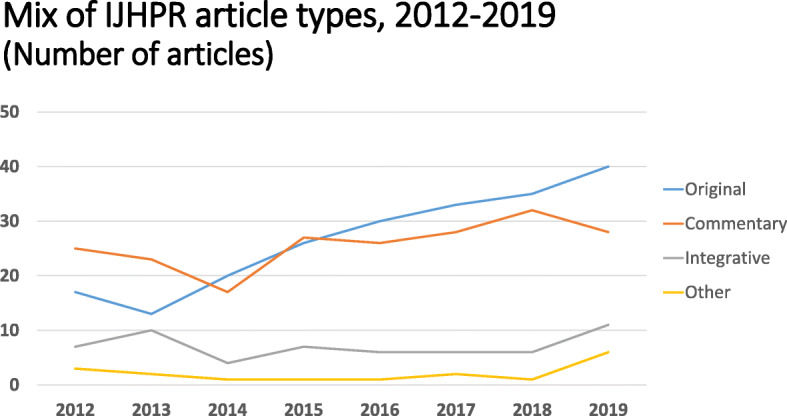
Mix of article types, 2012–2019 (Number of articles). Original, Commentary, Integrative article, Other

As shown in Table 1, the number of published articles increased from 52 in 2012 to 85 in 2019, an increase of 63% over seven years. There was also a change in the mix of article types. In 2012, original research articles accounted for about a third of all IJHPR articles and commentaries accounted for about half of them. This situation reversed in 2019, when the original research articles had risen to about half of the articles published, and the share of the commentaries had declined to about one-third. In terms of the number of articles, the large increase in the number of original research articles (from 17 to 40) accounted for most of the overall increase from 52 to 85. As indicated in Fig. 1 this major increase in the number of original research articles reflects a long-term trend at the IJHPR.

The topical mix of the articles exhibited both stability and change. “The health care workforce” and “health promotion/disease prevention” were leading topics in both 2012 and 2019. In contrast, both “quality of care” and “the patient-physician interaction” figured prominently in the 2012 topical mix, but were much less significant in the 2019 topical mix. Conversely, “mental health” and “health disparities” figured much more prominently in the 2019 mix than in the 2012 mix.

Looking to the future, we would like to see the topical mix of the IJHPR continue to evolve, along with the evolution of policy changes and opportunities facing Israel and other countries. For example, we expect that we will be receiving and publishing more articles related to digital health in the future. Other topics which are becoming more prominent include continuity of care and knowledge translation.

Table 2 indicates that the number of original research articles published by the IJHPR increased from 17 in 2012 to 40 in 2019. In both years, the authors used survey data (either solely or in part) in 53% of the original research articles. Authors’ use of databases (in whole or in part) increased slightly – from 35 to 43%.

The percentage of studies that involved secondary analysis of existing data also increased from 35 to 43%. Strikingly, almost all of these involved datasets from operational databases and only one of them involved secondary analysis of survey data.

Interestingly, the percentage of original research articles that involved analysis of changes over time declined from 24 to 13%.

The rationale behind our interest in changes over time, the use of databases, and the secondary use of survey data, can be found in Table 3.

**Table 3:**
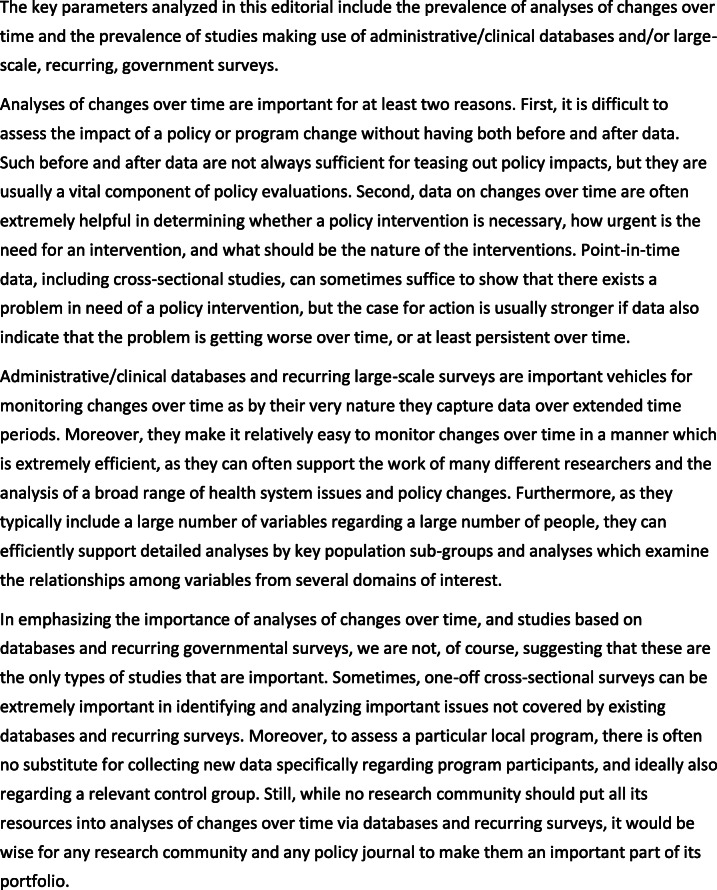
The rationale for key analyses undertaken in this editorial

Looking to the future, we would like to see the IJHPR publishing more articles that examine changes over time, as these can often provide uniquely important input into health policy decisions. We hope to work with research funders and academic institutions to encourage more publishable research that examines changes over time.

### Drilling down regarding the 2019 research articles

In the sections that follow, we take a closer look at the 40 original research articles that the IJHPR published in 2019. We first look at an issue related to article objectives – how the various articles sought to contribute to health policy. We then move on to issues related to methods, and in doing so we look separately at articles based on administrative or clinical databases and articles based on surveys.

### How the IJHPR’s 2019 original research articles sought to contribute to health policy

Many different types of studies can contribute to the development of health policy. Of the 40 original research articles published by the IJHPR in 2019, 50% (20 articles) sought to do so by examining one or more aspects of performance of a longstanding component of the Israeli health care system or of the health system as a whole. Another 28% (11 articles) sought to contribute to health policy development by analyzing population needs. Only 13% (5 articles) sought to do so via analyzing the impact of the initiation of, or changes in, national policies or programs, and only 5% (2 articles) sought to do so via analyzing the impact of institutional (i.e. hospital or health plan) programs.^4^ The remaining 2 original articles did not entail original research about the Israeli health system: one was a study of a health care program in Germany and the other was methodological/conceptual in nature.

### Further information on the 2019 articles that relied on databases

In 2019, the IJHPR published 17 original research articles that made use of existing databases. Israel’s Ministry of Health, including sub-units such as the Gertner Institute for Epidemiology and Health Policy Research and the Israel Center for Disease Control, was the database owner for 10 of these articles. Maccabi Healthcare Services^5^ and the Hadassah Medical Organization were each the database owners for 2 of the articles, and the Ministry of Defense, Sha’are Zedek Medical Center, and Terem^6^ were each the database owners for one of the articles. In 16 of these articles, there was at least one author from within the organization that owns the relevant database. In 11 of those the lead author is, or was, from that organization. Four out of 17 of the database articles included at least one author not from the organization that owns the relevant database. In short, investigators from outside the owning organization are occasionally involved in articles based on databases, but almost always in conjunction with investigators from within the owning organization and rarely as the lead authors.

The following are examples of 2019 articles which made use of databases:

- Two articles by investigators from the MOH’s Gertner Center, in which they used the Israel National Trauma Registry to explore Arab-Jewish differences in trauma incidence, care, and outcomes. One of the articles observed ethnic disparities between Jewish and Arab hospitalized casualties observed with regard to hospital stay, ICU admission and rehabilitation transfer [3]. The other article found that ethnic differences occur in violence-related injuries and recommended that violence prevention programs should be culturally adapted [4].
- An article by investigators from the Ministry of Health (MOH) and elsewhere used an MOH database on hospitalizations and procedures to explore how hysterectomy rates vary across regions and how they changed between 2007 and 2016 [5]. It found a decline in rates which are apparently due in part to the implementation of new technologies allowing earlier diagnosis, minimally invasive surgery, and alternative, non-surgical treatment modalities.
- An article by investigators from Maccabi Healthcare Services and elsewhere used Maccabi’s member database, delineated patient and physician’s characteristics related to high utilization of gastroenterology services, found regional differences, and recommended further training of primary care physicians in gastroenterology to improve the care and decrease the cost of managing patients with GERD (Gastro-Esophageal Reflux Disease) [6].
- An article by investigators from Hadassah that used Hadassah’s patient care database showed a substantial increase in use of mechanical ventilation and outlined budgetary, operational, and staffing consequences for hospitals and the entire healthcare system that need to be addressed [7].

Table 4 provides examples of publicly accessible government data sets. Some of these are based on surveys, while others are based on administrative/clinical databases. The agencies responsible for them include the Ministry of Health, the Israel Center for Disease Control, and the Central Bureau of Statistics. Almost none of these were used in IJHPR articles published in 2019 and they constitute fertile opportunities for future research and publication which are open to all. Significantly, some of these publicly available databases relate not to people but to organizations such as health plans, or to organizational units such as hospital departments. Several others have data about people, but rather than having data about individual people they have data at the aggregate level, such as locality. In the US, and probably in other countries as well, both empirical studies of organizations and studies based on aggregate data play an important role in health services research.

**Table 4:** Examples of publicly accessible governmental databases with multi-year data on health or health care, by agency responsible

**ICDC surveys:**	
• INHIS - Israel National Health Interview Survey	
• KAP - Knowledge Attitudes and Practices Survey	
• MABAT – Nutrition Surveys	
**MOH annual reports on the following:**	
• Health plan financial statements	
• Healthcare workforce	
• Hospital utilization statistics	
• ED utilization statistics	
**MOH – Internet BI on the following:**	
• Patient experience surveys (hospitals – general, psych, rehab, EDs, LTC)	
• OECD comparisons – health status, utilization, expenditure, financing, etc.	
• Hospital quality (from 2016)	
• Health status and service availability by locality	
• Patient complaints	
**Central Bureau of Statistics:**	
• National health expenditures	
• Household expenditure surveys	
• Labor force surveys	
• Social survey	
• Vital statistics (including life expectancy and causes of death)	
**National Insurance Institute**	
• Health plan membership reports	

Looking to the future, it would be good if Israeli funders and academic institutions explored ways to encourage more such research by Israeli investigators. The IJHPR and its co-editors would be happy to play a role in any such efforts.

We also note that the need to improve access to institutional databases was a major focus of the 16th Dead Sea Health System Leadership Conference in 2016 [8]. There was a consensus among conference participants that health information is a national resource. There was also a consensus regarding the value of promoting scientific research and population health through collaboration in the use of information. The conference participants recommended that health information be shared on the basis of reciprocity and equality, while maintaining the privacy of patients and safeguarding the legitimate interests of the organizations possessing the databases. The conference further recommended that steps be taken to promote the accessibility of databases, including accessibility to researchers. Traditionally, the conference recommendations are not binding, but they are seriously considered, and many of them are adopted.

Unfortunately, to date these recommendations from 2016 have not been sufficiently translated into policy. We reiterate the conference recommendations and call for prompt action to address this issue.

### Further information on the 2019 articles which relied on surveys

In 2019 the IJHPR published 21 articles which made use of survey data. 17 of the 21 were based solely on survey data, while 4 of the 21 were based on a combination of survey data and administrative/clinical databases. Only 1 of the 21 articles that made use of survey data involved secondary analysis of a prior survey. It was an article on the relationship between health disparities and socioeconomic status and it made use of the CBS Social Survey [9].

The number of respondents varied greatly across the 21 surveys. Three of them had 1000 or more respondents, 6 had fewer than 100 respondents, and 12 had between 100 and 1000 respondents.

Almost all the surveys used in 2019 IJHPR articles involved one-off cross-sectional surveys carried out specifically for the study being presented. None of the survey-based articles were used to examine changes over time.

Examples of 2019 IJHPR articles based on surveys include:

- An analysis of factors associated with childhood influenza vaccination in Israel, by a team from the ICDC, based on a survey of a national representative sample of 1040 adults meeting study criteria [10].
- A study of new mothers’ preferences for mental health treatment for post-partum depression, by a team from BGU, based on a survey of 1000 mothers who attended an MCH clinic for their infant’s first medical exam [11].
- A study of factors affecting oral examinations and dental treatments among older adults in Israel, by an investigator from The Max Stern Yezreel Valley College, based on a survey of a sample of adults aged 50–75, using probability sampling of groups defined by socio-demographic characteristics [12].

Looking to the future, we plan to publish fewer articles based on small samples and non-representative samples. We would also be happy to work with funders and academic institutions to encourage more secondary analyses of publicly available data from large-scale surveys by governmental agencies and other organizations. These surveys are almost always well-designed and well-executed. Moreover, most of them are carried out periodically, facilitating analyses of changes over time, which are so important for evaluating past policy interventions and for designing new interventions.

Of course, there will always be a place for one-off surveys on important issues, and some of these will be valuable even if they rely on relatively small samples. Our point is about the mix of surveys, and we would like to see greater reliance on large-scale surveys, particularly those that are carried out periodically and enable analysis of trends, in general, or pre-post policy changes. We also want to emphasize the importance of ensuring that the survey-based research be based on administration of the surveys to representative samples of the population to maximize the probability that the results are valid and generalizable.

### Additional information and insights

Appendix A presents preliminary information on IJHPR’s 2020 article mix, the mix of articles published by Israelis in other health policy and services journals, the types of articles which figure prominently in key comparator journals, and possible explanations for the findings suggested by experts in Israel and abroad.

It indicates that the key findings of the analysis of the IJHPR’s 2019 article mix also hold true for the IJHPR in 2020 and for the articles being published more generally by Israelis in related journals. In contrast, it illustrates how articles in key comparison journals publish more assessments of national policy interventions, appear to place more emphasis on changes over time, and tend to be based more on secondary analyses of large, publicly funded and publicly available surveys.

Conversations with past and present scientific directors of the National Institute for Health Policy (NIHP) and several past and present leaders of Israeli schools of public health suggest that several factors are probably contributing to the differences noted between journals and between countries. These include differences between countries in:

- How research is funded;
- The extent to which scholars have access to important administrative databases;
- The nature of the relationships between government and research institutions;
- The extent to which government carries out ongoing, large-scale surveys related to health care;
- The process for determining what will be covered in governmental surveys; and
- The opinions of academics about what constitutes a master’s thesis and doctoral dissertation;
- Academic promotion criteria
- The research culture.

Several of the experts emphasized the difficulty in Israel in accessing key governmental and institutional databases for research purposes. They noted that while this situation is improving, particularly in some of the institutions, there is still important work to be done in this area.

## Conclusions

We are proud of the articles being published by the IJHPR. At the same time, we feel that there are important opportunities to broaden and enrich the journal, as well as Israeli health services research more generally.

As the senior editors of the IJHPR we are interested in working with funders, academic institutions, the owners and principal users of key administrative databases, and individual scholars to further understand the factors influencing the mix of research being carried out, and subsequently published, by Israel’s health services research community. We would like the quality of submissions to IJHPR to reflect the same standards that lead Israeli authors to submit their work to other journals.

We believe that it will be important to analyze the mix of studies funded by the NIHP, the mix of abstracts presented at NIHP conferences, and the mix of articles related to Israel health services and health policy that appear anywhere in the Web of Science. Discussions with key players could also contribute to a deeper understanding of the findings and their causes. This deeper understanding could then be used to develop a joint plan to diversify and enrich Israel’s efforts in the fields of health policy analysis and health services research.

## Data Availability

Not applicable.

## Appendix

### Additional information and insights

This appendix consists of an initial look at the IJHPR’s 2020 article mix, the mix of articles published by Israelis in other health policy and services journals, the types of articles which figure prominently in other key journals in the field. It also presents possible explanations for the findings, as suggested by experts in Israel and abroad.

### An initial look at the IJHPR’s 2020 set of articles

As 2020 is still in progress, we were unable to analyze the full set of 2020 articles. Instead, we assessed whether the mix of the original articles published in January–October 2020 (*N* = 37) was similar to the mix of the full set of articles published in 2019, in terms of key parameters considered in this editorial. We found that, as in 2019, about 40% of the studies made use of databases. In addition, as seen in 2019, about a third of the 2020 articles based on databases examined changes over time. Finally, as in 2019 almost none of the articles based on surveys looked at changes over time. In fact, out of 22 articles using survey data, only two of them used surveys to look at changes over time [13] [14], and only one of them made secondary use of data from an existing survey [14].

### Selected findings regarding Israeli articles in related journals

It could very well be that some of the findings presented above regarding the IJHPR do not reflect the full set of health services and health policy articles published by Israeli authors. This could be the result of a tendency of many Israeli authors to submit what they think is their very best work to journals whose impact factors are higher than the IJHPR’s 1.74.^7^

To begin to explore this issue, we carried out a preliminary analysis of the 50 articles with Israeli authors that were published in 2019 in any of the 86 journals, aside from the IJHPR, in the Web of Science’s Health Policy and Services category. We found that 14 (28%) made use of existing databases and that 7 (14%) examined changes over time. We also found that only 1 (2%) made secondary use of existing surveys, and only 4 (8%) consisted of assessments of existing or potential national policy interventions or programs.

The national policy interventions or programs assessed were: national reforms in mental health and social care services [15], financial incentives to attract medical residents to the periphery [16], and the health promoting schools network [17], and a potential policy of government sharing of financial risks with health plans [18].^8^

The only article in the collection to employ secondary analysis of an existing data set used data from the Survey of Health, Ageing and Retirement in Europe (SHARE) at two selected time points (2006/2007 and 2015) to investigate changes in the rate of informal care provision, which occurred in Israel and Italy in the last decade [19].

To be sure, many Israeli health services / health policy articles are being published in journals in other WoS categories, and particularly the Public, Environmental, and Occupational Health category. It could well be that Israeli articles in that category have a greater tendency to examine changes over time and to make secondary use of large scale, national surveys. However, it is unlikely that many of those articles relate to personal health services and policy interventions related to those services. Empirical studies should be carried out to assess these conjectures.

### Types and examples of relevant articles being published in key comparator journals

A quick look at the table of contents of recent issues of Health Services Research, Health Policy, and Health Affairs, suggests that, in comparison with the IJHPR, these journals publish more assessments of national policy interventions and they appear to place more emphasis on changes over time. Their articles also tend to be based more on secondary analyses of large, publicly funded and publicly available surveys. Moreover, their surveys (even the one-off surveys) tend to be larger.^9^

For example, on the website of Health Policy, the January 2020 issue is the most recent issue that is fully publicly available. The two lead articles are about Finland’s 2018 Road to Alcohol Act [20], and Canada’s 2018 decision to establish an Advisory Council on adding pharmaceuticals to universal health coverage [21].

The Health Affairs website features several articles from their September 2020 issue. The first one listed is “Medicaid Expansion Improved Perinatal Insurance Continuity for Low-Income Women”. Its abstract states “We used survey data from the 2012–17 Pregnancy Risk Assessment Monitoring System to estimate the impact of Affordable Care Act–related state Medicaid expansions on continuity of insurance coverage for low-income women across three time points: preconception, delivery, and postpartum” [22].

The lead article in the October 2020 issue of Health Services Research is entitled “Reducing the prevalence of low-back pain by reducing the prevalence of psychological distress: Evidence from a natural experiment and implications for health care providers”. It made secondary use of data from 1998 to 2004 from three different U.S. national surveys: The National Health Interview Survey, The National Ambulatory Medical Care Survey, and The National Hospital Ambulatory Medical Care Survey [23].

In conjunction with the preparation of this editorial, we held several discussions with leading scholars from abroad who are familiar with a broad range of health services and health policy journals. They confirmed that our impressions based on the three journals noted above also hold true more widely for leading journals in our field.

## Abbreviations

IJHPR: *Israel Journal of Health Policy Research*
ICDC: Israel Center for Disease Control
MOH: Ministry of Health
NIHP: National Institute for Health Policy

## Footnotes

1 The IJHPR is in the second quartile in the Journal Citation Report’s public, environmental, and occupational health category, and in the third quartile in the JCR’s health policy and services category. In the public, environmental, and occupational health category, it is at the 53rd percentile (80/170), and in the health policy and services category it is at the 40th percentile (53/87).

2 While most of the journal’s empirical articles are written by Israeli authors and focus on Israeli health care, about one-half of the IJHPR’s articles have included at least one author from a non-Israeli institution. Many of these are commentaries that either highlight the international significance of an Israeli study or use the international experience to suggest possible next steps for Israel. In addition, authors from abroad sometimes collaborate with Israeli authors on empirical studies focused on Israeli health care or on studies comparing Israel with one or more countries. Overall, the IJHPR has published approximately 50 papers involving collaborations between an author based primarily at an Israeli institution and an author based at a non-Israeli institution [1].

3 .

4 Interestingly, of the 11 integrative articles the IJHPR published in 2019, 2 explored the impact of national program or policy changes and 1 explored the impact of a health plan program change.

5 Maccabi is Israel’s second largest health plan, with over 2 million members

6 Terem is a network of emergency medical centers
